# Longitudinal Gut Microbiota Dysbiosis Underlies Olanzapine-Induced Weight Gain

**DOI:** 10.1128/spectrum.00058-23

**Published:** 2023-06-01

**Authors:** Li Qian, Xiaoyan He, Yixin Liu, Fengjie Gao, Wen Lu, Yajuan Fan, Yuan Gao, Wei Wang, Feng Zhu, Yanan Wang, Xiancang Ma

**Affiliations:** a Department of Psychiatry, The First Affiliated Hospital of Xi’an Jiaotong University, Xi’an, China; b Center for Brain Science, The First Affiliated Hospital of Xi’an Jiaotong University, Xi’an, China; c Shaanxi Belt and Road Joint Laboratory of Precision Medicine in Psychiatry, The First Affiliated Hospital of Xi’an Jiaotong University, Xi’an, China; d Center for Translational Medicine, The First Affiliated Hospital of Xi’an Jiaotong University, Xi’an, China; e Med-X institute, Center for Immunological and Metabolic Diseases, The First Affiliated Hospital of Xi'an Jiaotong University, Xi’an, China; China Agricultural University

**Keywords:** schizophrenia, second-generation antipsychotics, metabolic disorders

## Abstract

Olanzapine is one of the most effective medicines available for stabilizing schizophrenia spectrum disorders. However, it has been reported to show the greatest propensity for inducing body weight gain and producing metabolic side effects, which cause a great burden in patients with psychiatric disorders. Since the gut microbiota has a profound impact on the initiation and development of metabolic diseases, we conducted a longitudinal study to explore its role in olanzapine-induced obesity and metabolic abnormalities. Female Sprague-Dawley rats were treated with different doses of olanzapine, and metabolic and inflammatory markers were measured. Olanzapine significantly induced body weight gain (up to a 2.1-fold change), which was accompanied by hepatic inflammation and increased plasma triglyceride levels (up to a 2.9-fold change), as well as gut microbiota dysbiosis. Subsequently, fuzzy c-means clustering was used to characterize three clusters of longitudinal trajectories for microbial fluctuations: (i) genera continuing to increase, (ii) genera continuing to decrease, and (iii) genera temporarily changing. Among them, *Enterorhabdus* (*r* = 0.38), *Parasutterella* (*r* = 0.43), and *Prevotellaceae* UCG-001 (*r* = 0.52) positively correlated with body weight gain. In addition, two MetaCyc metabolic pathways were identified as associated with olanzapine-induced body weight gain, including the superpathway of glucose and xylose degradation and the superpathway of l-threonine biosynthesis. In conclusion, we demonstrate that olanzapine can directly alter the gut microbiota and rapidly induce dysbiosis, which is significantly associated with body weight gain. This may suggest gut microbiota targets in future studies on metabolic abnormalities caused by olanzapine.

**IMPORTANCE** Olanzapine is one of the most effective second-generation antipsychotics for stabilizing schizophrenia spectrum disorders. However, olanzapine has multiple drug-induced metabolic side effects, including weight gain. This study provides insight to the gut microbiota target in olanzapine-induced obesity. Specifically, we explored the longitudinal gut microbiota trajectories of female Sprague-Dawley rats undergoing olanzapine treatment. We showed that olanzapine treatment causes a dynamic alteration of gut microbiota diversity. Additionally, we identified three genera, *Parasutterella*, *Enterorhabdus*, and *Prevotellaceae* UCG-001, that may play an important role in olanzapine-induced obesity. In this case, the supply or removal of specific elements of the gut microbiota may represent a promising avenue for treatment of olanzapine-related metabolic side effects.

## INTRODUCTION

Atypical antipsychotic drugs (AAPDs), such as olanzapine, are second-generation antipsychotics currently used to treat schizophrenia and other chronic psychotic disorders, largely because of their low incidence of extrapyramidal adverse effects (1). Unfortunately, AAPDs have multiple drug-induced metabolic side effects, including weight gain, dyslipidemia, and impairments in glucose homeostasis (2, 3). These metabolic side effects are associated with a long-term increased risk of diabetes mellitus, ischemic heart disease, and overall mortality (4). Olanzapine, one of the most clinically effective AAPDs (5, 6), is also associated with greater weight gain and other disorders involved in the development of metabolic syndrome (7, 8). Given that unwanted metabolic side effects compromise compliance with long-term treatment regimens and quality of life (9), it is important to explore the mechanisms underlying these side effects.

Although the mechanisms by which olanzapine causes metabolic side effects are still controversial, they are partly attributed to its diverse pharmacological receptor profile (10). Olanzapine has been shown to significantly increase liver lipid accumulation by increasing the expression of mammalian target of rapamycin complex-1 in rats (11). In addition, olanzapine induces hyperphagia associated with the development of obesity in female rats through hypothalamic histamine H1 receptor and AMP-activated protein kinase signaling (12, 13). Moreover, olanzapine-induced weight gain and metabolic disturbances are associated with polymorphisms in the 5-hydroxytryptamine 2C receptor (14, 15). There seems to be a correlation between the increased clinical efficacy of olanzapine and the increased risk of metabolic side effects, which is attributed to same-acting receptors (16).

Recent studies indicated that in addition to acting on diverse receptors, olanzapine may affect the gut microbiota (17), which has a profound impact on the body weight, metabolism, and systemic inflammation of the host (18). For instance, in germfree mice, the gut microbiota is necessary and sufficient for weight gain caused by oral olanzapine delivery (19). Moreover, olanzapine-induced metabolic dysfunction is attenuated by antibiotic administration in rats (20, 21). Although previous studies have demonstrated that olanzapine treatment can affect the gut microbiota in addition to affecting body weight, metabolism, and systemic inflammation (21), few studies have clarified its exact effect on microbiota composition and function. In our previous experiment, olanzapine did not successfully mimic the effects of human weight gain in mice. Previous studies have shown that the effect of olanzapine on body weight can be effectively modeled in female Sprague-Dawley (SD) rats (22, 23). Similarly, clinical studies have shown that olanzapine treatment increased the risk of obesity in women (24, 25). Here, we conducted a longitudinal study to identify the dynamic effect of olanzapine on the gut microbiota and investigated the correlations between these changes and body weight changes in female SD rats.

## RESULTS

### Olanzapine treatment markedly increases body weight and body weight gain without affecting food intake.

**FIG 1:**
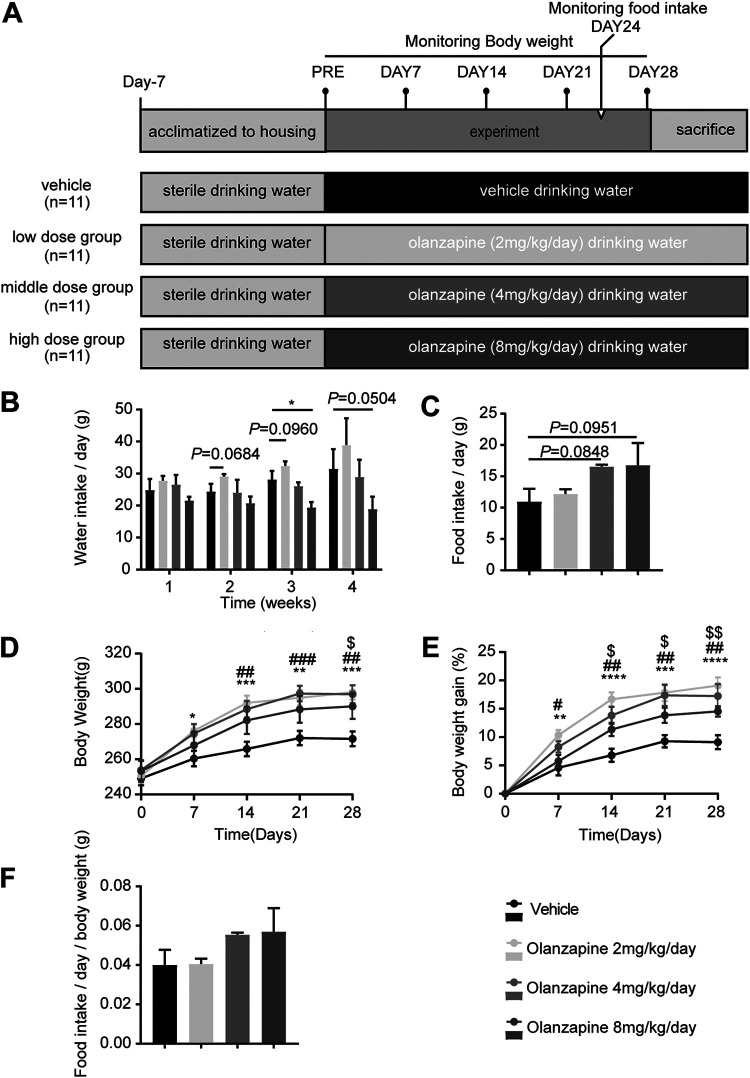
Olanzapine treatment causes body weight gain without affecting food intake. (A) Female Sprague-Dawley rats, weighing approximately 250 g, were fed a chow diet and received vehicle treatment or three doses of olanzapine (2, 4, or 8 mg/kg body weight per day) via drinking water for 4 weeks (*n* = 11). (B) Rats were housed with 2 to 3 animals per cage, and the total water intake of individual cages was measured and divided by the number of rats in each cage to obtain the average water intake per rat (*n* = 9). (C) Food intake was measured the same way as water intake in the fourth week of intervention (*n* = 9). (D to F) Body weight was measured weekly (D), and the percentages of body weight gain (E) and average food intake per body weight (F) were calculated. Data are shown as the means ± standard error of the mean. Olanzapine 2 mg/kg versus vehicle groups: *, *P < *0.05; **, *P < *0.01; ***, *P < *0.001; ****, *P < *0.0001. Olanzapine 4 mg/kg versus vehicle groups: *#*, *P < *0.05; #*#*, *P < *0.01; ##*#*, *P < *0.001. Olanzapine 8 mg/kg versus vehicle groups: $, *P < *0.05; $$, *P < *0.01.

Olanzapine treatment induced a significant change in water intake (F [3, 8] = 10.16, *P = *0.0042) (Fig. 1B). Compared with the vehicle-treated group, rats treated with olanzapine at a dose of 2 mg/kg body weight/day showed a tendency to increase water intake at week 2 (*P = *0.0684) and 3 (*P = *0.0960), while rats treated with olanzapine at a dose of 8 mg/kg/day displayed clearly decreased water intake at week 3 (*P = *0.0140) and 4 (*P = *0.0504). In contrast, none of the olanzapine-treated groups exhibited any significant alteration in food intake (F [3, 8] = 2.038, *P = *0.187) (Fig. 1C). In addition, we observed that the three doses of olanzapine (2, 4, or 8 mg/kg/day) all significantly increased body weight (F [3, 40] = 3.042, *P = *0.0398) and body weight gain (F [3, 40] = 9.129, *P < *0.0001) after 4 weeks of treatment (Fig. 1D and E). There was a significant interaction between treatment and intervention time (F [12, 160] = 6.667, *P < *0.0001). Rats administered olanzapine at doses of 2 or 4 mg/kg/day showed increased body weight evident from the first week that persisted through the whole intervention period, whereas rats treated with olanzapine at a dose of 8 mg/kg/day displayed significant weight gain from week 2. In line with nonadjusted food intake, olanzapine treatment did not affect the body-weight-adjusted food intake (Fig. 1F).

### Olanzapine treatment increased liver weight and plasma triglyceride (TG) level.

**FIG 2:**
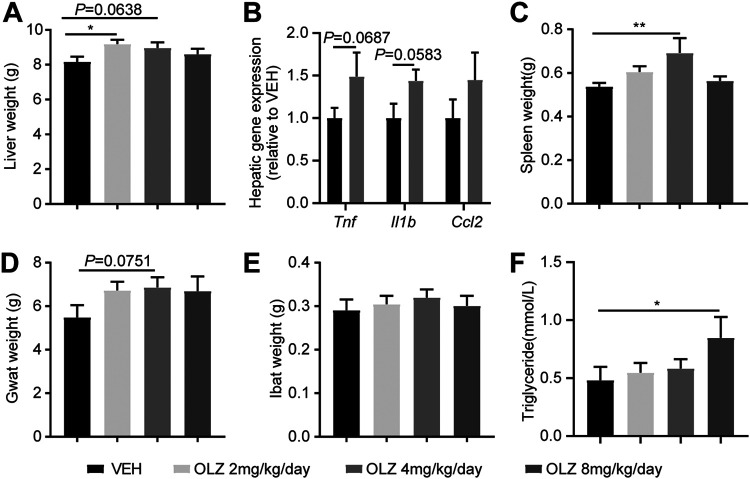
Olanzapine treatment increases liver weight and plasma triglyceride levels. (A to E) After 4 weeks of intervention, rats were sacrificed via cervical dislocation, organs were collected immediately, and the weights of the liver (A), spleen (C), gonadal white adipose tissue (D), and interscapular brown adipose tissue (E) were determined. The relative mRNA expression of *Tnf*, *Il1b*, and *Ccl2* in the liver was measured using real-time qPCR (B). (F) The triglyceride plasma levels were determined via commercially available enzymatic kits. Data are shown as means ± standard error of the mean (*n* = 11 rats per group); *, *P < *0.05; **, *P < *0.01 compared with the vehicle group.

Compared with the vehicle-treated group, olanzapine treatment at the dose of 2 mg/kg/day significantly increased liver weight (*P = *0.0187), and at the dose of 4 mg/kg/day tended to increase liver weight (*P = *0.0638; Fig. 2A). Given that olanzapine has been shown to predispose individuals to a proinflammatory state (26, 27), we next determined the expression levels of inflammation-related genes in the livers of olanzapine- and vehicle-treated rats. Compared with the vehicle group, the hepatic expression levels of the proinflammatory markers tumor necrosis factor-α (TNF-α) (*Tnf*; *P = *0.0687) and interleukin-1β (IL-1β) (*Il1b*; *P = *0.0583) were clearly upregulated in the olanzapine-treated group (4 mg/kg/day; Fig. 2B). Consistently, olanzapine treatment at a dose of 4 mg/kg/day significantly increased the weights of the spleen (*P = *0.0074; Fig. 2C) and gonadal white adipose tissue (gWAT) (*P = *0.0751; Fig. 2D), without affecting the interscapular brown adipose tissue (iBAT) (Fig. 2E). Moreover, we observed that olanzapine treatment at a dose of 8 mg/kg/day significantly elevated plasma TG levels (*P = *0.0383; Fig. 2F).

### Olanzapine treatment induced dynamic gut microbiota dysbiosis.

**FIG 3:**
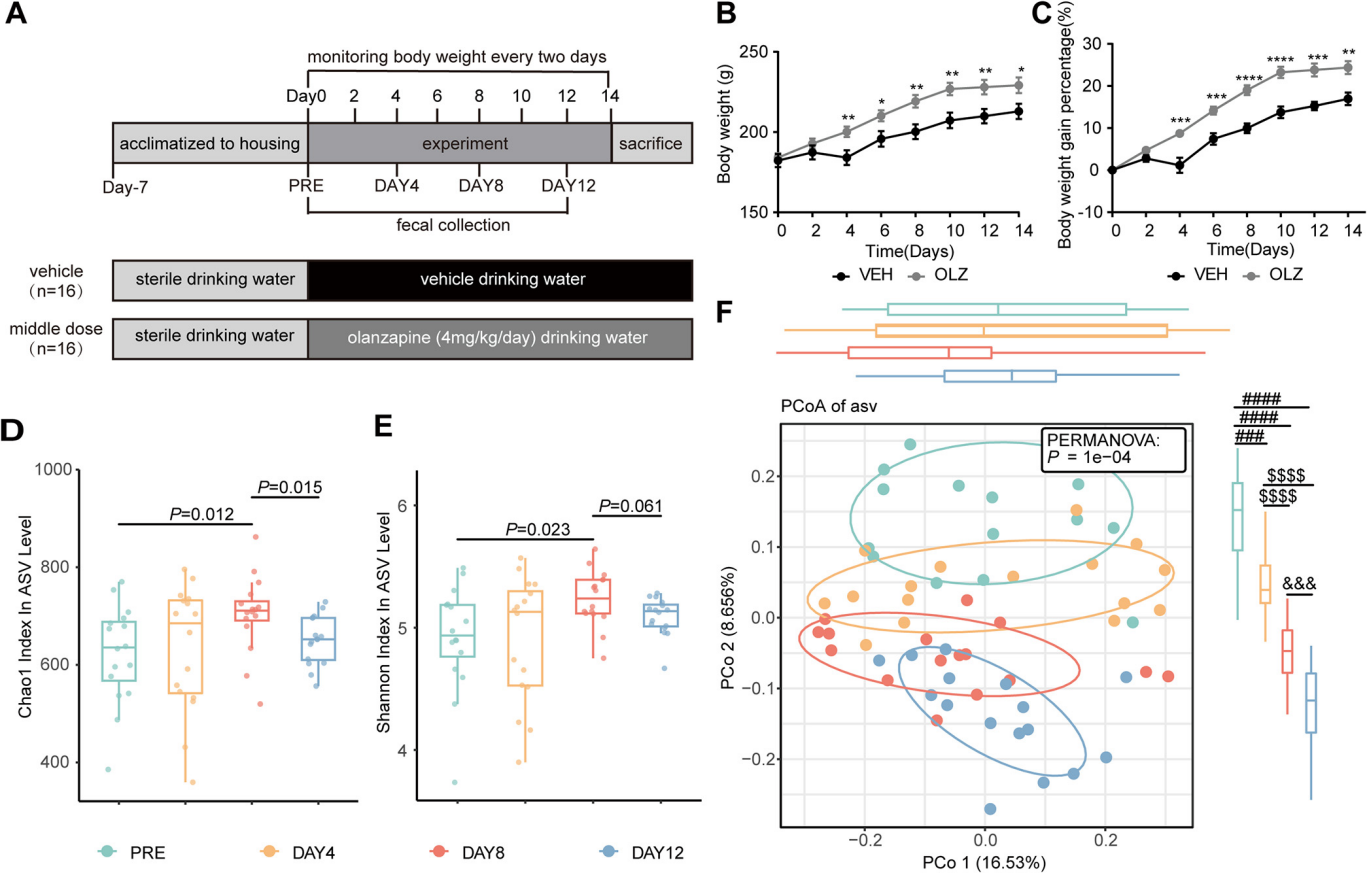
Olanzapine treatment causes a dynamic alteration of gut microbiota composition. (A) Female Sprague-Dawley rats, weighing approximately 180 g, were fed a chow diet and received vehicle or olanzapine (4 mg/kg/day) treatment via drinking water for 12 days. (B and C) Body weight was measured every 2 days (B), and the percentage of body weight gain was calculated (C). Fresh fecal samples were collected every 4 days, and total bacterial DNA was isolated for 16S rRNA sequencing. (D to F) At baseline (PRE) and after olanzapine intervention on days 4, 8, and 12, Chao1 diversity (D) and Shannon diversity (E) at the amplicon sequence variant level were assessed, and principal-coordinate analysis (F) was constructed based on the Bray=Curtis matrix. The 25th to 75th percentiles of the distribution are represented by a box, the median of the distribution is indicated by the line in the middle of the box, and outliers of the distribution are represented by dots. Data are shown as means ± standard error of the mean (*n* = 16 rats per time point). Olanzapine 4 mg/kg versus vehicle groups: *, *P < *0.05; **, *P < *0.01; ***, *P < *0.001; ****, *P < *0.0001. compared with PRE: ##*#*, *P < *0.001; ###*#*, *P < *0.0001. Compared with day 8: $$$$, *P < *0.0001; &&&, *P < *0.001.

Next, we used longitudinal analysis to identify olanzapine-treatment-induced dynamic changes in the gut microbiota. Additionally, to minimize the influence of food or water intake on the gut microbiota and mimic the weight gain effect of olanzapine, we chose a dose of 4 mg/kg/day for the second study. In line with the first study, olanzapine at a dose of 4 mg/kg/day significantly increased the body weight (F [1, 30] = 6.088, *P = *0.0195) (Fig. 3B) and body weight gain (F [1, 30] = 25.09, *P < *0.0001) (Fig. 3C). An increase in rat body weight and body weight gain was already evident on the fourth day of treatment. Food intake was also tracked. In line with the first study, olanzapine treatment did not affect the body-weight-adjusted food intake (see Fig. S1 in the supplemental material). We analyzed the gut microbiome from fecal samples using 16S rRNA sequencing. On average, 84,071 (standard deviation, 2,828) reads per sample were generated; 99.94% of these sequences had a length of 399 to 428 bp, suggesting that most of the reads were valuable. The total reads were clustered into 6,416 amplicon sequence variants (ASVs) for further analysis. In both rarefaction curves of observed ASVs and Shannon diversity, all sequenced samples reached the plateau stage, representing a good sampling depth (Fig. S2A and B).

Host homeostasis relies heavily on the gut microbial community diversity. Therefore, we observed the effects of olanzapine on gut microbiota diversity. Fecal samples collected at different times had markedly different communities. Alpha diversities measured using the Chao1 and Shannon indexes changed significantly over time (Fig. 3D and E). The Chao1 and Shannon indexes markedly changed on day 8 (*P*_Chao1_ = 0.012, *P*_Shannon_ = 0.023) and recovered on day 12 (*P*_Chao1_ = 0.015, *P*_Shannon_ = 0.061). Principal-coordinate analysis (PCoA) showed that the beta diversities of the gut microbiota significantly separated among the baseline (PRE), day 4, day 8, and day 12 groups (*P < *0.0001) (Fig. 3F). The composition of the gut bacterial community differed significantly between the PRE and day 4 groups (*P = *0.010), PRE and day 8 groups (*P < *0.0001), and PRE and day 12 groups (*P < *0.0001). The mean relative abundance at the phylum and genus levels for each time point is shown in stacked bar charts (Fig. S3). The stacked bar chart at the phylum level showed that the sum of average relative abundance of two phyla, *Firmicutes* and *Bacteroidota*, accounted for more than 90% of the gut microbiota in rats before and after olanzapine treatment. The relative abundance of *Firmicutes* increased significantly from 45% ± 13% to 53% ± 6% (*P = *0.0216) following olanzapine treatment, while the relative abundance of *Bacteroidota* decreased significantly from 50% ± 14% to 42% ± 7% (*P = *0.0320). Compared with the PRE group, the ratio between *Firmicutes* and *Bacteroidota* (F/B ratio) in the day 8 group increased significantly (*P* = 0.0342). At all four time points, *Prevotella*, *Muribaculaceae*, and *Lactobacillus* ranked as the top three-most-abundant taxa enriched in the microbiota. The mean relative abundances of dominant genera were 19.9% for *Prevotella*, 13.7% for *Muribaculaceae*, and 5.3% for *Lactobacillus* in the PRE group and 12.8% for *Muribaculaceae*, 11.6% for *Prevotella*, and 8.4% for *Lactobacillus* in the day 12 group.

### Olanzapine treatment led to distinct changes in different gut microbiota subgroups over time.

**FIG 4:**
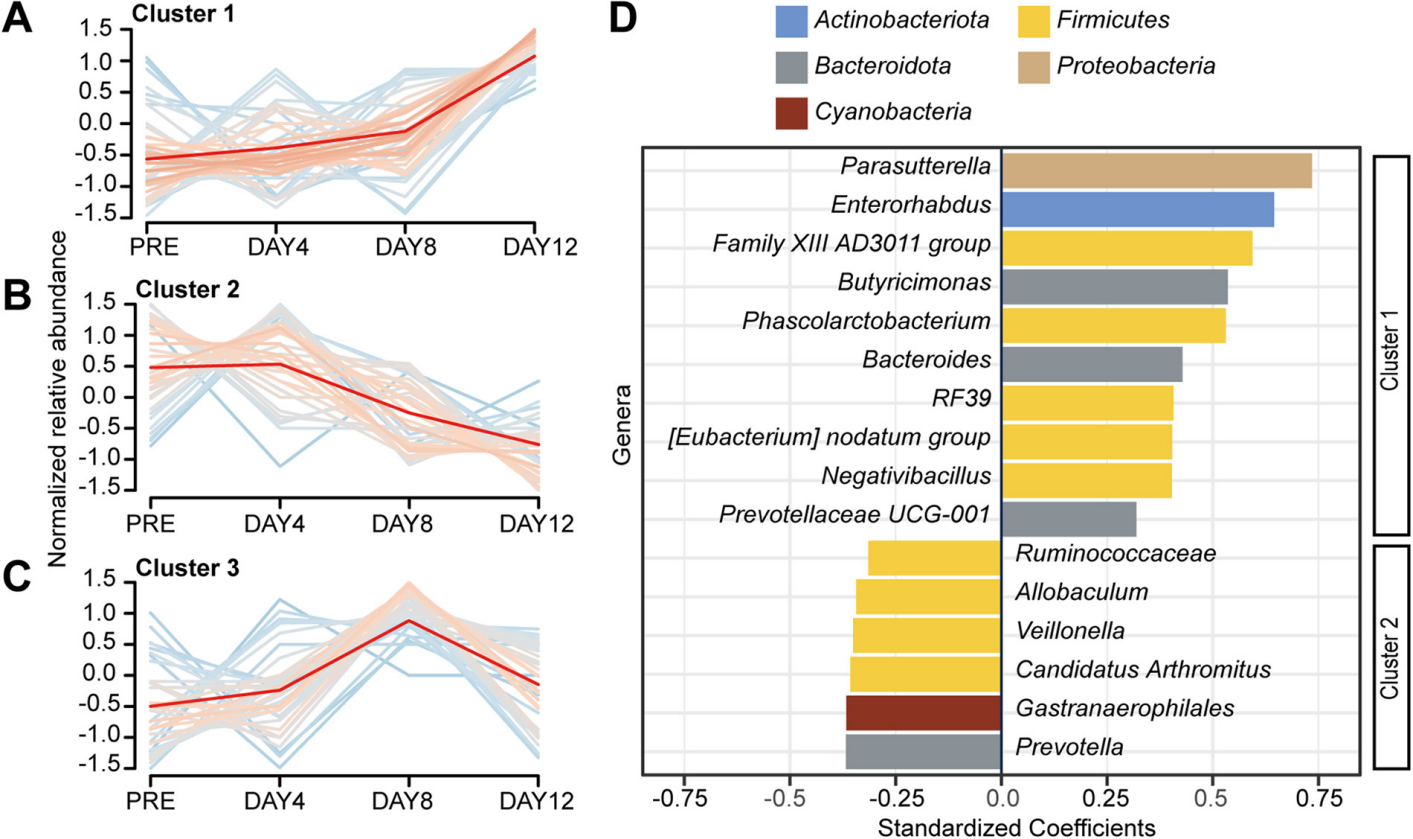
Olanzapine treatment leads to distinct changes in different subgroups of the gut microbiota over time. Fuzzy c-means clustering was used to classify the gut microbiota into different clusters based on longitudinal tracks. (A to C) The mean relative abundance of bacteria at the genus level was used to establish the longitudinal trajectories of three clusters. Detailed data for the genera in each cluster are shown in Fig. S3. Multiple linear regression analysis adjusted by cage was then performed between the gut microbiota and time. (D) A horizontal bar plot shows the standard coefficients for different genera.

To identify the different longitudinal trajectories of the gut microbiota in rats treated with olanzapine, we calculated the within-cluster sum of squares for different cluster numbers based on the mean relative abundance of the microbial genera. Three clusters of longitudinal trajectories for microbial fluctuation were identified via the “elbow” method (Fig. S4A and B). Then, the longitudinal trajectories of the three clusters were constructed using fuzzy c-means clustering, including (i) genera continuing to increase from PRE to day 12 (*n* = 45; Fig. 4A); (ii) genera continuing to decrease from PRE to day 12 (*n* = 50; Fig. 4B), and (iii) genera temporarily changing from PRE to day 12 (*n* = 45; Fig. 4C). `Detailed information on the gut microbiota of each cluster is shown in Fig. S5. To verify the correlation between gut microbiota and time, genera in clusters 1 and 2 with relative abundances of >0.01% were selected to construct multivariate linear regression models. After adjusting for cages, 16 genera were found to have changed significantly over time. The abundance of the [*Eubacterium*] *nodatum* group (*P = *0.0014), *Enterorhabdus* (*P < *0.0001), *Bacteroides* (*P = *0.0008), Family XIII AD3011 group (*P < *0.0001), *Butyricimonas* (*P < *0.0001), *Negativibacillus* (*P = *0.0016), *Parasutterella* (*P < *0.0001), RF39 (*P = *0.0018), *Phascolarctobacterium* (*P < *0.0001), and *Prevotellaceae* UCG-001 (*P = *0.0151) increased significantly over time (Fig. 4D). Conversely, the abundance of *Allobaculum* (*P = *0.0101), *Prevotella* (*P = *0.0036), “*Candidatus* Arthromitus” (*P = *0.0074), *Ruminococcaceae* (*P = *0.0161), *Gastranaerophilales* (*P = *0.0061), and *Veillonella* (*P = *0.0067) decreased significantly over time (Fig. 4D).

### Body weight gain correlated with longitudinal changes in gut microbiota induced by olanzapine treatment.

**FIG 5:**
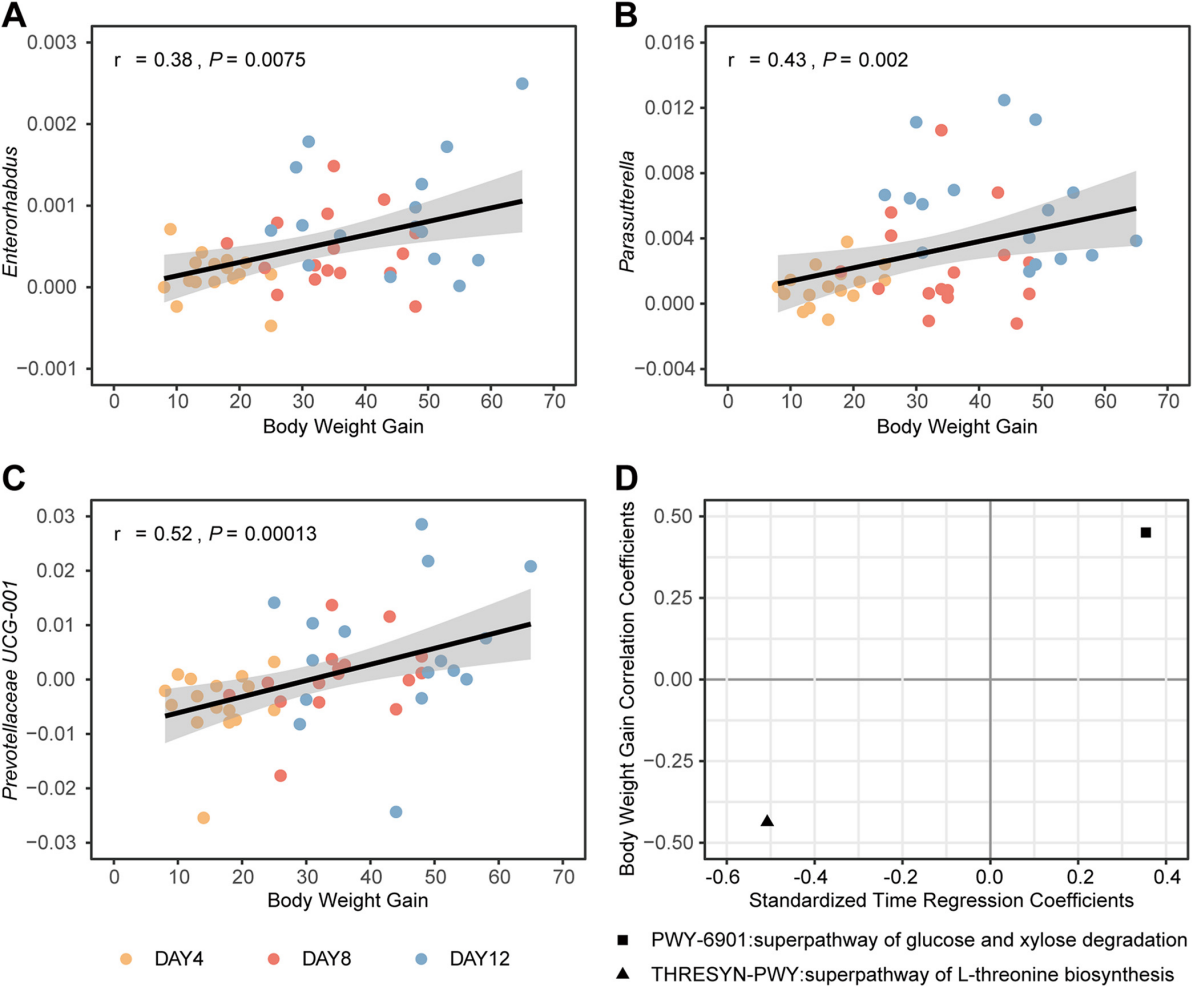
Body weight gain correlates with longitudinal changes of gut microbiota induced by olanzapine treatment. Spearman’s correlation was calculated between gut microbiota changes and body weight gain. (A to C) Scatterplots with linear regression lines were used to show these correlations. (D) Linear mixed models and Spearman’s correlation were also conducted in phylogenetic investigation of communities by reconstruction of unobserved states-predicted MetaCyc pathways to determine time-dependent and weight-gain-associated metabolic pathways.

The above-described evidence shows the sizeable effect of olanzapine on gut microbiota; however, it is still unclear which genera play an essential role in the increased body weight gain caused by olanzapine. To identify the body weight-associated genera, Spearman’s correlation was applied to integrate gut microbiota changes and body weight gain in the olanzapine-treated group. Three genera strongly associated with body weight gain were identified as *Enterorhabdus* (*r* = 0.38, *P = *0.0075), *Parasutterella* (*r* = 0.43, *P = *0.002), and *Prevotellaceae* UCG-001 (*r* = 0.52, *P = *0.00013) (Fig. 5A to C). Additionally, the metabolic pathways of the gut microbiota were predicted using PCRUSt2 in the MetaCyc database, and a multivariable linear regression model was constructed to determine the time-dependent metabolic function. A total of 70 MetaCyc metabolic pathways passed the hypothesis test, with false-discovery rate (FDR)-corrected *P* values < 0.1 in the regression model. Spearman’s correlation was then used to identify the metabolic pathways associated with weight change. Notably, olanzapine treatment significantly altered two metabolic pathways of the gut microbiota, the superpathway of glucose and xylose degradation (*r* = 0.45, *P = *0.0013) and the superpathway of l-threonine biosynthesis (*r* = –0.44, *P = *0.0019), both of which were significantly correlated with body weight change (Fig. 5D).

## DISCUSSION

To the best of our knowledge, this is the first study to show rapid dynamic changes in the gut microbiota accompanied by substantial body weight gain upon olanzapine treatment, implying that the gut microbiota may play a critical role in olanzapine-induced obesity. Patients with schizophrenia, major depressive disorder, or bipolar disorder who take olanzapine are at a high risk of obesity (28), with the underlying mechanism remaining unclear. Currently, clinical treatment strategies for olanzapine-related obesity include nonpharmacological (e.g., optimizing exercise and diet) and pharmacological (e.g., metformin) interventions. However, those strategies are limited by poor adherence or insufficient effectiveness (3, 16, 17). The discovery of new mechanisms paves the way for further potential treatments. The gut microbiota has recently been identified as playing an important role in obesity development (29). In the present study, we demonstrated, through longitudinal analysis of female specific-pathogen-free (SPF) SD rats combined with fuzzy c-means clustering, multivariable linear regression, and Spearman correlation, that olanzapine may cause obesity by affecting the abundance of *Enterorhabdus*, *Parasutterella*, and *Prevotellaceae* UCG-001.

Previous studies have confirmed that female SD rats are excellent models for mimicking olanzapine-induced weight gain (22). The terminal half-life of olanzapine is very short in rats (2.5 h) compared with that in humans (21 to 54 h) (30, 31). Therefore, to ensure the exposure of rats to olanzapine during the day and night and to facilitate timely adjustment of drug concentration according to body weight, we opted for drinking water administration. The olanzapine dose range was selected based on information provided in previous literature. Olanzapine at the dose of 7.5 mg/kg/day reflects a 70% dopamine D2 receptor occupancy in rats, a threshold associated with an optimal chance of antipsychotic efficacy in humans (32). In a rat model, olanzapine at the dose of 2 mg/kg/day has been demonstrated to produce plasma concentrations roughly equivalent to the human therapeutic range (33). In this study, olanzapine at doses of 2, 4, and 8 mg/kg/day induced rapid weight gain in female rats, consistent with a previous report (34). We did not observe a dose-dependent effect of olanzapine treatment on body weight gain. This corresponds with previous research showing that lower doses are not necessarily associated with a smaller weight gain (35, 36). Previous studies reported that an increase in appetite plays a key role in olanzapine-induced weight gain (37). However, in our study, although a modest trend of increased food intake was observed in the olanzapine-treated group at the 4 and 8 mg/kg doses, after adjustment for body weight, none of olanzapine treatments affected food intake levels. The results of our study suggest that olanzapine-treatment-induced weight gain is likely not connected with varied food intake.

Consistent with previous studies (38, 39), we observed that olanzapine treatment caused significantly increased weights of the liver, spleen, and white adipose tissue. In addition, the livers of rats treated with olanzapine displayed a marked increase in TNF-α and IL-1β expression. This suggests that olanzapine treatment can predispose individuals to a proinflammatory state. According to a previous study, immunomodulatory transcriptional networks are enriched in mice prone to olanzapine-related weight gain (40). It has been shown that olanzapine significantly changes the structure of the spleen and increases TNF-α levels (27) and that long-term olanzapine treatment in rodents elevates the plasma levels of proinflammatory cytokines (41). Furthermore, we observed that olanzapine treatment significantly increased plasma TG levels, consistent with previous reports (39, 42). Increased visceral mass is considered a key factor in the development of metabolic syndrome, especially insulin resistance (43). Our findings suggest that olanzapine-induced obesity, and in particular, the liver, spleen, and white adipose tissue weight gain, may lead to metabolic inflammation and impaired lipid and glucose metabolism. Furthermore, metformin has been shown to prevent olanzapine-induced metabolic dysfunction and regulates the gut-liver axis in rats (26).

In recent years, longitudinal study designs have gained popularity. Compared with single-time-point studies, they provide more information on periodic patterns or interdependencies within the microbiome (44). In the model exploration experiment, we found that the weight gain caused by olanzapine increased most rapidly in the first and second weeks and reached a plateau in the third and fourth weeks. In order to determine the impacts of the gut microbiota on weight gain, we collected fresh feces continuously in the first 2 weeks after olanzapine treatment. We observed that olanzapine treatment induced a dynamic gut microbiota dysbiosis, evident from day 4 of treatment, and led to distinct changes in different gut microbiota subgroups over time, suggesting the sizeable effects of olanzapine on the gut microbiota. Body weight changes were consistent with the patterns of changes in gut microbiota diversity, suggesting the importance of the overall gut microbiota composition in olanzapine-induced body weight gain. Previous studies have shown gut microbiota dysbiosis as an important contributor to obesity (28, 29), and the *Firmicutes*/*Bacteroidota* ratio plays an important role in obesity development (45, 46). We observed that olanzapine treatment increased the *Firmicutes*/*Bacteroidota* ratio, accompanied by body weight gain. Antibiotic-induced microbiome depletion eliminated olanzapine-induced body weight gain (20). Taking probiotics or prebiotics is one effective way to change the gut microbiota diversity (47, 48). The probiotic mixture VSL#3 reverses olanzapine-induced metabolic dysfunction in rodents (49). Probiotics or probiotics plus dietary fiber can attenuate antipsychotic-induced weight gain in patients (50, 51). However, a human study also showed that olanzapine intervention did not influence the gut microbiota in a cohort composed of 20 patients. Because of the ability to standardize environments, to precisely quantify food intake, and to perform certain manipulations, the gut microbiota in model animals is less affected by confounding factors from the environment than that of humans (52). Our preclinical study indicates that olanzapine may directly impact gut microbiota composition, accompanied with host metabolic changes.

We observed three longitudinal trajectories for the disturbed gut microbiota. The gut microbiota in clusters 1 and 2 showed a relatively stable change trend. Among these, the changes of *Enterorhabdus*, *Parasutterella*, and *Prevotellaceae* UCG-001 were significantly correlated with body weight gain. According to a previous study, *Parasutterella* is positively associated with body mass index, fasting insulin, and type 2 diabetes, independent of low-grade inflammation (53). This large European and Canadian cohort study indicates a role of *Parasutterella* in human obesity by affecting the fatty acid biosynthesis pathway (53). GMrepo is a database that links human phenotypes and gut microbiota (54). According to GMrepo, *Parasutterella* was prevalent in patients with prehypertension (78.6%) and hypertension (69%). *Enterorhabdus* positively correlated with obesity-related parameters and negatively correlated with hepatic low-density lipoprotein receptor expressions (55). *Prevotellaceae* UCG-001 was found to be decreased in ob/ob (leptin-deficient) mice (56). Taken together, these findings show that it is plausible that olanzapine-induced gut microbiota dysbiosis may contribute to the body weight gain, hepatic inflammation, and increased plasma triglyceride levels, while dedicated studies will be needed to determine the causality. A previous study has confirmed that the vagus nerve is essential for olanzapine-induced body weight gain (38). One possible explanation is the gut microbes affected by olanzapine treatment may act on the vagus nerve to regulate total body energy metabolism, which could be investigated by experiments combining gut microbe translation with vagotomy surgery. In addition, we observed that olanzapine treatment decreased the abundance of *Akkermansia*, which has been shown to improve olanzapine-induced glucose homeostasis in mice (57). Cluster 3 contained genera that changed only briefly and then returned to the baseline level over time. However, several genera in this cluster were related to metabolic disorders in previous studies (58, 59), which may have been a result of short-term fluctuations in gut microbiota.

In addition, two MetaCyc metabolic pathways were found to be altered by olanzapine intervention in this study and were significantly associated with changes in body weight. The gut microbiota is known to influence host homeostasis, in part via various metabolites (60). On one hand, the superpathway of glucose and xylose degradation can produce lactate, which plays important roles in inducing insulin resistance in systemic and local organs (61), deteriorating cardiometabolic disease (62), and metabolic syndrome (63). On the other hand, the superpathway of l-threonine biosynthesis was downregulated by olanzapine intervention; l-threonine plays a beneficial role in improving metabolic abnormalities and obesity (64, 65). However, the PICRUSt2 approach used to predict gut microbiota metabolic activity should be considered with caution, and metagenomic analyses should be conducted in humans to verify these findings. These studies indicate that olanzapine treatment may induce gut microbiota dysbiosis and rapidly alter gut microbiota metabolism, as a result promoting obesity.

In summary, we showed that olanzapine has significant effects on several metabolic, inflammatory, and microbial parameters. Through a longitudinal study, we observed a direct effect of olanzapine on the gut microbiota *in vivo*. Our findings indicate that olanzapine may have induced dynamic changes in the gut microbiota, which could contribute to olanzapine-mediated body weight gain. The functional role and mode of action of the identified gut microbiota are still not clear, and future studies are required to clarify the underlying mechanisms.

## MATERIALS AND METHODS

### Animals.

Female SPF SD rats, initially weighing approximately 200 g, were purchased from the Laboratory Animal Center of Xi’an Jiaotong University; they were group-housed (3 to 4 rats per cage) to avoid the stress caused by single housing. The animals had free access to a standard chow diet (Xietong Shengwu, Jiangsu, China) and sterile water. All rats were acclimatized to standard conditions (20 to 26°C; 12/12 h light/dark cycle) for 7 days and randomized based on body weight to groups receiving olanzapine (Oulanning, Hansoh Pharma, Jiangsu, China) or vehicle (0.1 M acetic acid and 0.1 M NaOH) via drinking water. In the first study, rats were treated with olanzapine (2 mg/kg/day), olanzapine (4 mg/kg/day), olanzapine (8 mg/kg/day), or vehicle for 28 days, and body weight and water intake were assessed weekly and food intake was assessed at week 4 (Fig. 1A; *n* = 11 rats per group). In the second study, rats were treated with olanzapine (4 mg/kg/day) or vehicle for 12 days, body weight was evaluated every other day, and fresh fecal pellets were collected every 4 days (Fig. 3A; *n* = 16 rats per group). The doses were selected to reflect therapeutic concentrations in patients and have been previously shown to induce weight gain and metabolic side effects in rats (32, 33). The sample size was also selected based on previous studies (66–68). All experiments were performed in accordance with the National Institutes of Health Laboratory Animal Care and Use Guidelines (NIH Publication no. 80-23) and were approved by the Department of Science, Technology, and Discipline Construction of the Xi’an Jiaotong University Health Science Center (2022-507).

### Sample collection and plasma lipid profiles.

Trunk blood was collected via heart puncture in EDTA-coated tubes and centrifuged for 10 min at 3,000 rpm. Plasma was derived from the supernatant and used for determining TG levels via commercially available enzymatic kits (Roche Diagnostics, Mannhein, Germany). The liver, spleen, gWAT, and iBAT were weighed using an electronic balance and frozen in a −80°C freezer for long-term storage and later analysis.

### RNA preparation and real-time quantitative PCR (qPCR).

Total RNA was extracted from the liver using TRIzol reagent (Invitrogen, California, USA). Then, the RNA was reverse-transcribed using the Evo M-MLV RT Premix for qPCR kit (Accurate Biotechnology, Hunan, China) in a C1000 Touch thermal cycler (Bio-Rad, California, USA) according to the manufacturer’s instructions. Target gene expression was detected via real-time qPCR using a SYBR green premix *Pro Taq* high-sensitivity (HS) qPCR kit (Accurate Biotechnology, Hunan, China) on a CFX 96 real-time PCR system (Bio-Rad, CA). The expression level of each gene was normalized to those of *B2m* and *Rplp0* and expressed as the fold change compared with the vehicle group. Primer sequences are shown in Table S1.

### Extraction of DNA from fecal samples and 16S rRNA sequencing.

Fecal pellets were collected directly from the animals at baseline (PRE) and after treatment at day 4, day 8, and day 12 on dry ice and quickly stored at −80°C. Total fecal bacterial DNA was extracted using an EZNA stool DNA kit (Omega Bio-Tek, Georgia, USA) according to the manufacturer’s instructions. The quality of the extracted bacterial genomic DNA was assessed using agarose gel electrophoresis. The highly conserved V3 to V4 region of the bacterial 16S rRNA gene was amplified via PCR using the TopTaq DNA polymerase kit (TransGen, Beijing, China). Universal 16S rRNA primers (forward primer, F 5′-CCTACGGGNGGCWGCAG-3′; reverse primer, R 5′-GACTACHVGGGTATCTAATCC-3′) with specific barcodes per sample were used for PCR amplification. The PCR conditions consisted of initial denaturation at 94°C for 2 min, followed by 25 cycles at 94°C for 30 s, 55°C for 30 s, and 72°C for 1 min, and a final extension at 72°C for 10 min. The PCR products were assessed using agarose gel electrophoresis, purified using Agencourt AMPure XP PCR purification beads (Beckman Coulter, California, USA), and quantified using an Invitrogen Qubit 3.0 spectrophotometer (Thermo Fisher Scientific, Massachusetts, USA). Amplicon pools were then prepared for sequencing, and the size and quantity of the amplicon library were assessed using an Agilent 2100 bioanalyzer. The libraries were sequenced on a NovaSeq 6000 platform (Illumina, California, USA) using an SP-Xp (PE250) double-ended strategy.

QIIME2 was used to remove possible adapter sequences and primers, filter data quality, and annotate ASVs (69). Finally, the sequences were phylogenetically assigned to taxonomic classifications using a Ribosomal Database Project (RDP) classifier (version 2.2) in accordance with the RDP (release 11.5). After phylogenetic allocation of the sequences down to the phylum, class, order, family, and genus levels, relative abundance was defined as the number of sequences of a given phylogenetic taxonomy divided by the total number of sequences per sample. Alpha and beta diversity analyses were performed using the ASV-level table. Rarefaction curves of the observed ASVs and Shannon-Wiener diversity were constructed to determine sequencing depth. The Chao1 estimator for community richness and Shannon index for community diversity were evaluated for each sample. The Bray-Curtis distance matrix based on the composition at the ASV level was calculated and subsequently used to perform PCoA. Permutational multivariate analysis of variance was performed to compare Bray-Curtis distances among groups. Functional shifts in the microbiota at different time points were predicted using PICRUSt2 according to the MetaCyc database (https://metacyc.org/) (70).

### Statistical analysis.

Statistical analyses were performed using Prism v7.0 (GraphPad, California, USA). Two-way repeated measure analysis of variance (ANOVA) was used to analyze body weight, body weight change, and water intake, with treatment and time as factors. One-way ANOVA was used for food intake, organ weight, and TG, with treatment as a factor. Statistical details of the ANOVA tests are provided in the results part: F(DFn, DFd), where F is the F value, DFn is the degrees of freedom of the numerator, and DFd is the degrees of freedom of the denominator. Multiple comparisons of data were performed using Fisher’s least significant difference test. Gene expression between two groups was evaluated using an unpaired two-tailed Student’s *t* test. Data are presented as means ± standard error of the mean. Statistical significance was set at *P < *0.05.

Microbial community analyses were performed using R software (version 4.2.1). To classify the longitudinal pattern of taxonomic changes in the gut microbiota, we calculated the within-cluster sum of squares for different cluster numbers and used the elbow method to obtain the optimal number of clusters (71). The results were assessed using the R package NbClust (72). Fuzzy c-means clustering was used to classify the different trajectories of the gut microbiota using the Mfuzz R package (73). Multivariable linear mixed models, including cages as covariates, were computed using the lm base function after log_10_-transformation and Z-score scaling of the data (73, 74). The Spearman correlation test was performed to identify genera associated with body weight gain among the time-dependent genera. Subsequently, linear mixed models and Spearman’s correlation were used to determine time-dependent and weight-gain-associated MetaCyc metabolic pathways. FDRs for gut microbiota analysis were computed using the Benjamini-Hochberg approach (significance was determined at FDR <0.1) (75).
