# Supplementary figures and images for: Longitudinal Gut Microbiota Dysbiosis Underlies Olanzapine-Induced Weight Gain
Source: Microbiol Spectr. 2023 Jun 1;11(4):e00058-23. doi: 10.1128/spectrum.00058-23 (PMC10433857; doi:10.1128/spectrum.00058-23)

**A**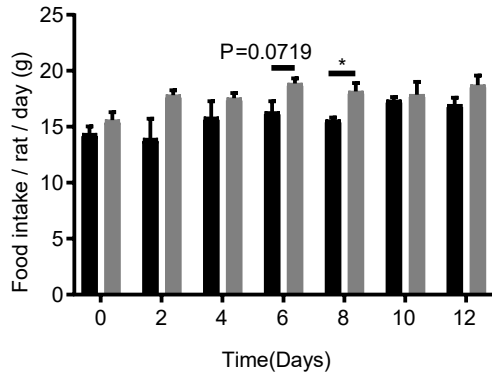**B**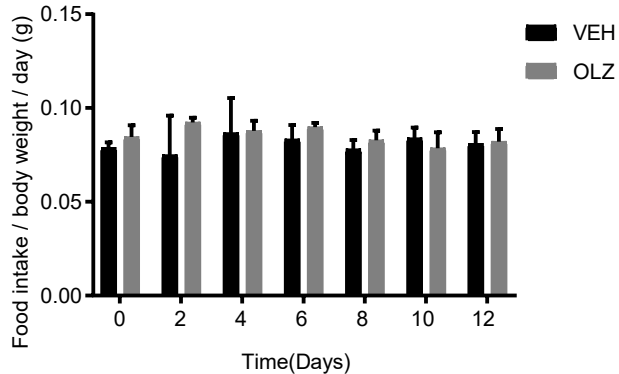

**Figure S1: Olanzapine treatment did not affect the body-weight-adjusted food intake.**

Supplement: Supplemental file 1 — Supplemental material. Download spectrum.00058-23-s0001.pdf, PDF file, 0.1 MB [file spectrum.00058-23-s0001.pdf]
