# Supplementary material for: Longitudinal Gut Microbiota Dysbiosis Underlies Olanzapine-Induced Weight Gain
Source: Microbiol Spectr. 2023 Jun 1;11(4):e00058-23. doi: 10.1128/spectrum.00058-23 (PMC10433857; doi:10.1128/spectrum.00058-23)
Supplement: Supplemental file 2 — Supplemental material. Download spectrum.00058-23-s0002.pdf, PDF file, 0.4 MB [file spectrum.00058-23-s0002.pdf]

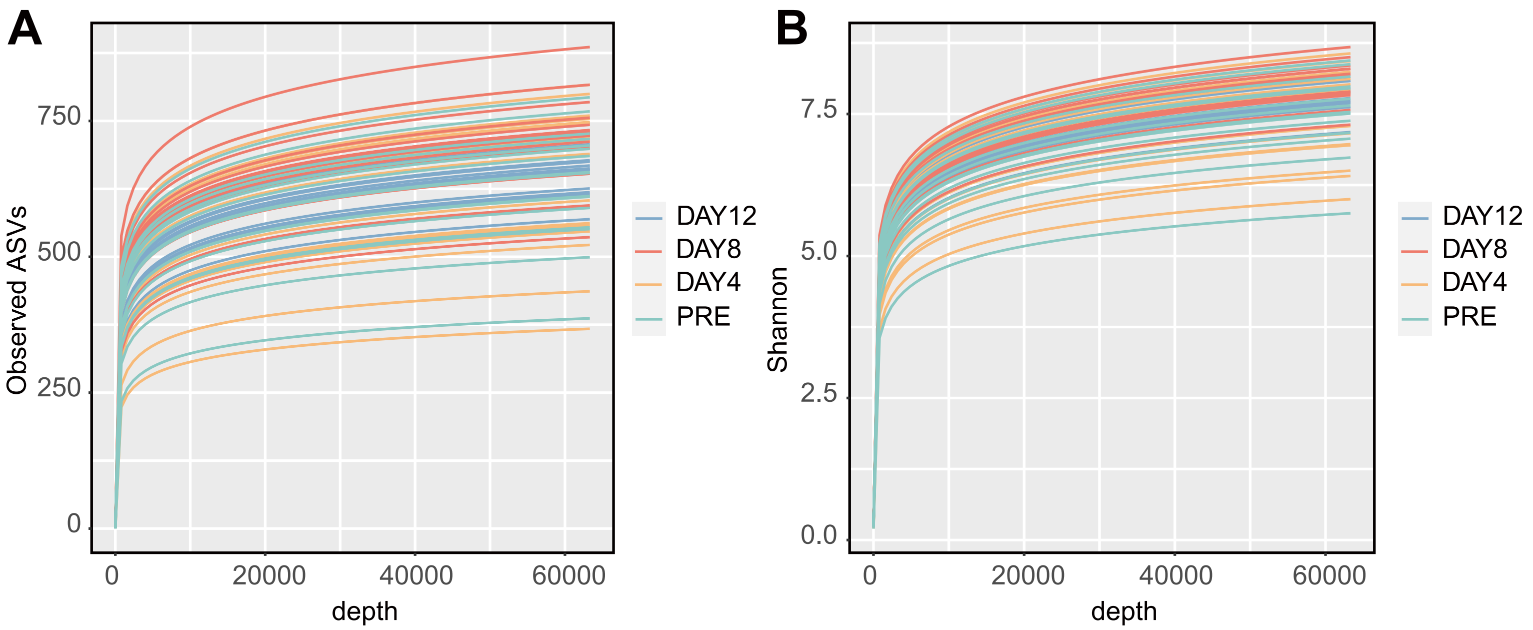

Figure S2: Rarefaction curves of observed amplicon sequence variants (ASVs) and Shannon–Wiener diversity for all samples. (A) Rarefaction curves of the observed ASVs from faecal samples of individual rats. (B) Rarefaction curves of Shannon–Wiener diversity in faecal samples from individual rats. The curves are coloured to indicate the samples at different time points.
