# Supplementary material for: Longitudinal Gut Microbiota Dysbiosis Underlies Olanzapine-Induced Weight Gain
Source: Microbiol Spectr. 2023 Jun 1;11(4):e00058-23. doi: 10.1128/spectrum.00058-23 (PMC10433857; doi:10.1128/spectrum.00058-23)
Supplement: Supplemental file 3 — Supplemental material. Download spectrum.00058-23-s0003.pdf, PDF file, 0.4 MB [file spectrum.00058-23-s0003.pdf]

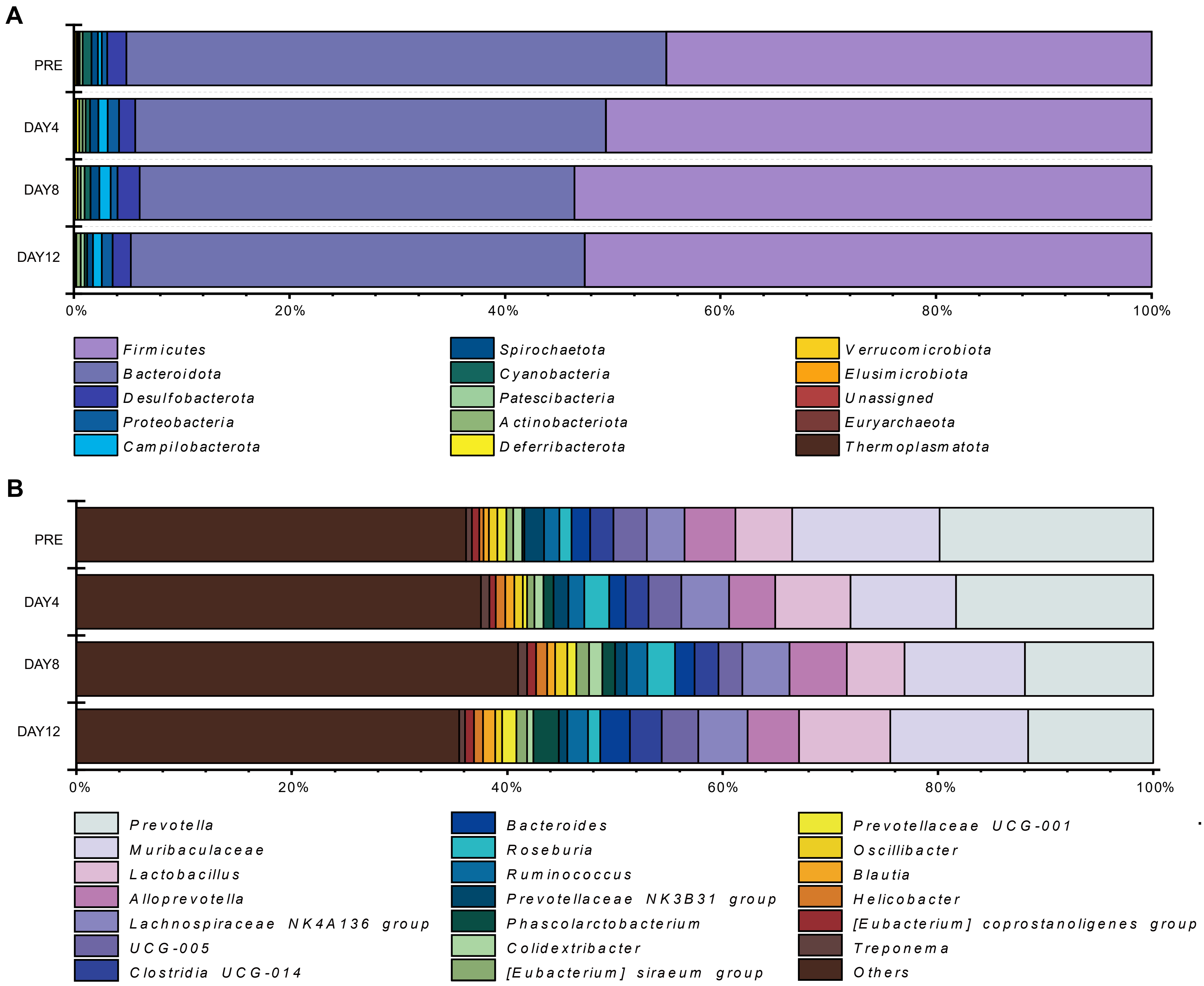

Figure S3: Olanzapine intervention increased the F/B ratio without altering the dominant gut microbiota at different time points. Stacked bar chart showing the mean relative abundance at the phylum (A) and genus (B) levels using RDP classification for PRE, DAY4,DAY8, and DAY12.
