# Supplementary material for: Longitudinal Gut Microbiota Dysbiosis Underlies Olanzapine-Induced Weight Gain
Source: Microbiol Spectr. 2023 Jun 1;11(4):e00058-23. doi: 10.1128/spectrum.00058-23 (PMC10433857; doi:10.1128/spectrum.00058-23)
Supplement: Supplemental file 4 — Supplemental material. Download spectrum.00058-23-s0004.pdf, PDF file, 0.2 MB [file spectrum.00058-23-s0004.pdf]

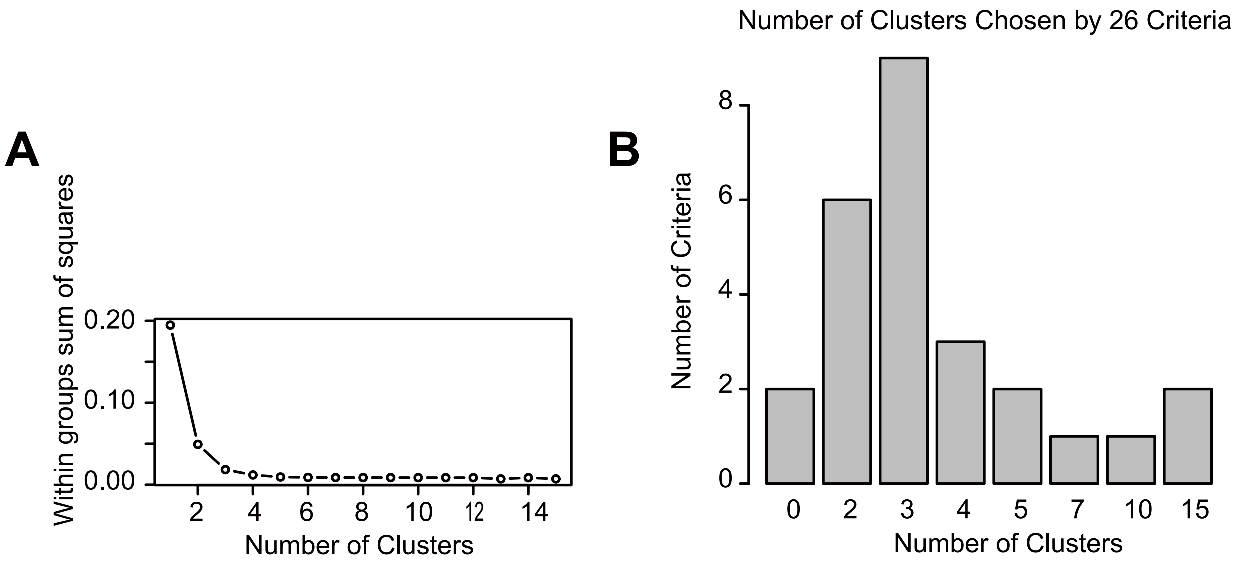

Figure S4: K-means clustering identifies three patterns of gut microbiota longitudinal change. The line chart displays the within-cluster sum of squares for the different clustering numbers (A). The slope of the segment indicates the influence of a change in the number of clusters on the within-cluster sum of squares. NbClust produced a histogram of up to 15 possible cluster groupings formed via k-means clustering (B). Atop each bar is the total number from the 26 criteria used to estimate the optimal cluster number.
