# Supplementary material for: Longitudinal Gut Microbiota Dysbiosis Underlies Olanzapine-Induced Weight Gain
Source: Microbiol Spectr. 2023 Jun 1;11(4):e00058-23. doi: 10.1128/spectrum.00058-23 (PMC10433857; doi:10.1128/spectrum.00058-23)
Supplement: Supplemental file 5 — Supplemental material. Download spectrum.00058-23-s0005.pdf, PDF file, 0.6 MB [file spectrum.00058-23-s0005.pdf]

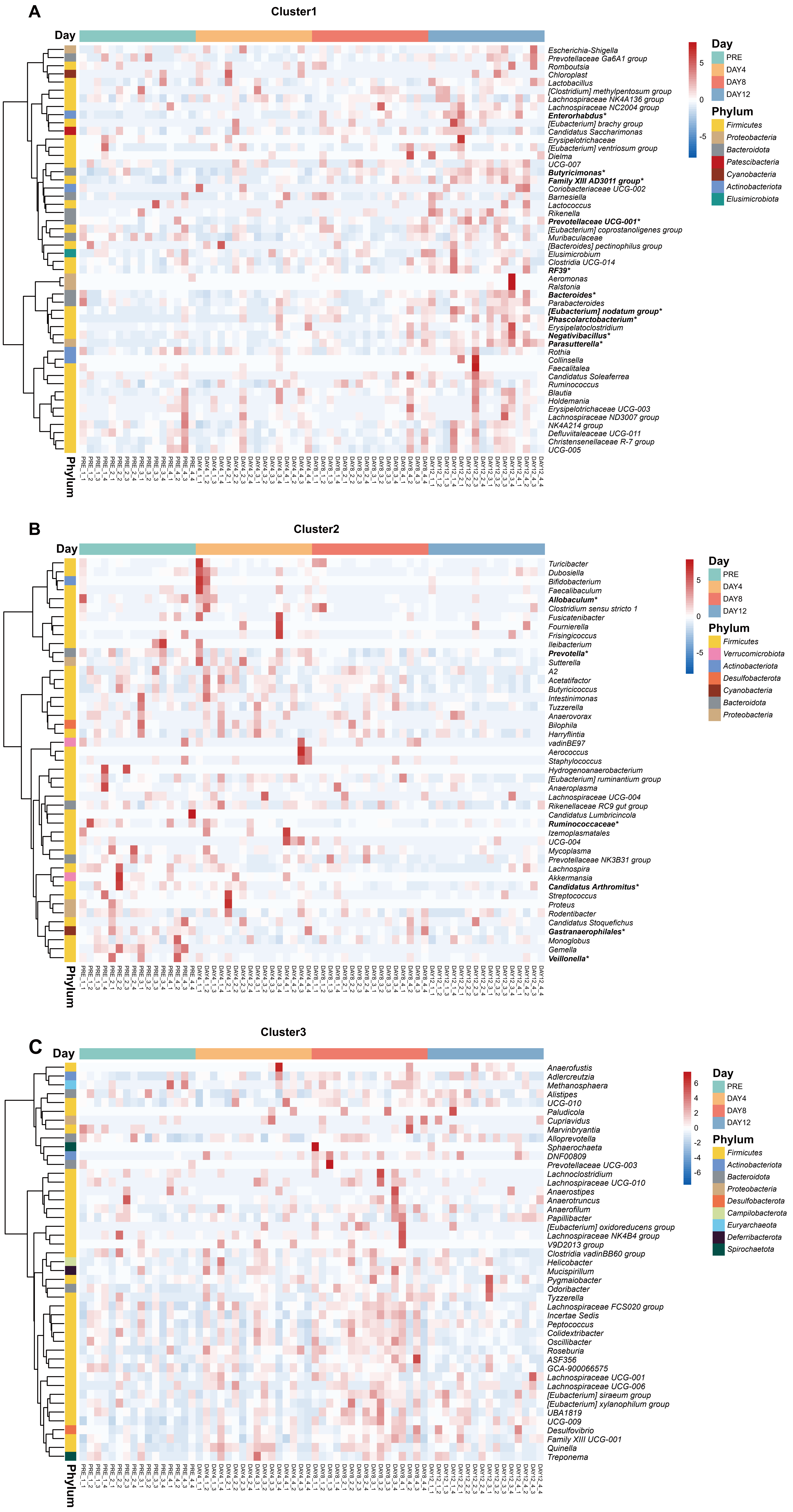

Figure S5: Olanzapine treatment causes disturbance of some gut microbiota. (A) Heatmap of hierarchical clustering of genera increase over time in Figure 4A. (B) Heatmap of hierarchical clustering of genera decrease over time in Figure 4B. (C) Heatmap of hierarchical clustering of genera temporary change over time in Figure 4C. Colour-coded heatmaps (blue to red) illustrate the row-scaled relative abundance of each genus in all samples. Horizontal annotation indicates different samples, with prefixes (PRE, DAY4, DAY8, DAY12) indicating different time points. The different coloured boxes on the left side of the heatmap indicate bacterial classification at the phylum level. “*” indicates either increase or decrease over time at false discovery rates <0.1, as calculated using multifactor linear regression.
