# Supplementary material for: Longitudinal Gut Microbiota Dysbiosis Underlies Olanzapine-Induced Weight Gain
Source: Microbiol Spectr. 2023 Jun 1;11(4):e00058-23. doi: 10.1128/spectrum.00058-23 (PMC10433857; doi:10.1128/spectrum.00058-23)
Supplement: Supplemental file 6 — Supplemental material. Download spectrum.00058-23-s0006.docx, DOCX file, 0.01 MB [file spectrum.00058-23-s0006.docx]

**Table S1:** **List of RT-qPCR primers used in this study.**

| **Gene name** | **Primer sequences(5'to3')** |
| --- | --- |
| *Tnf* (TNF-α) (F) | ACACACGAGACGCTGAAGTA |
| *Tnf* (TNF-α) (R) | GGAACAGTCTGGGAAGCTCT |
| *Il1b* (IL-1 β) (F) | GCACAGTTCCCCAACTGGTA |
| *Il1b* (IL-1 β) (R) | ACACGGGTTCCATGGTGAAG |
| *Ccl2* (MCP-1) (F) | GATCTCTCTTCCTCCACCACTAT |
| *Ccl2* (MCP-1) (R) | GTTCTCCAGCCGACTCATTG |
| *B2m* (F) | CGAGACCGATGTATATGCTTGC |
| *B2m* (R) | GTCCAGATGATTCAGAGCTCCA |
| *Rplp0* (F) | GAACATCTCCCCCTTCTCCTTC |
| *Rplp0* (R) | ATTGCGGACACCCTCTAGGAA |
